# Morphological study of the antennal sensilla in Gerromorpha (Insecta: Hemiptera: Heteroptera)

**DOI:** 10.1007/s00435-017-0354-y

**Published:** 2017-04-28

**Authors:** A. Nowińska, J. Brożek

**Affiliations:** Department of Zoology, Faculty of Biology and Environmental Protection, University of Silesia, Bankowa 9, 40-007 Katowice, Poland

**Keywords:** Morphology, Heteroptera, Antennae, Sensilla, Gerromorpha

## Abstract

The external morphology and distribution of the antennal sensilla of 21 species from five families of semiaquatic bugs (Gerromorpha) were examined using scanning electron microscopy. Nine main types were distinguished based on their morphological structure: sensilla trichoidea, sensilla chaetica, sensilla leaflike, sensilla campaniformia, sensilla coeloconica, sensilla ampullacea, sensilla basiconica, sensilla placoidea and sensilla bell-mouthed. The specific morphological structure of one type of sensilla (bell-mouthed sensilla) was observed only in *Aquarius paludum*. Several subtypes of sensilla are described, differentiated by number, location and type of sensillum characteristic for each examined taxon. The present study provides new data about the morphology and distribution of the antennal sensilla in Gerromorpha.

## Introduction

Gerromorpha, or semiaquatic bugs, are predatory insects that are associated with water. They live on the surface of the water, near water reservoirs or on aquatic plants (Andersen 1982). This taxon consists of 1900 species in eight families (Andersen 1982; Schuh and Slater 1995; Cardé and Resh 2012).

The antennae of Gerromorpha belong to the same morphological type as those found in other heteropteran insects. The scapus and pecicel are one antennomer while the flagellum consists of four antennomers (Andersen 1982; Schuh and Slater 1995). A significant part of the sensory system of insects consists of a large number of highly diverse organs called sensilla. These sensory organs are located in the antennae, mouthparts (labium, labial and maxillary palps and proboscis (a food-sucking tubular appendage), genitalia, legs and wings (Peregrine 1972; Backus 1985; Brożek and Bourgoin 2013; Brożek 2013; Brożek and Zettel 2014; de Bruyne and Baker 2008; Guerenstein and Hildebrand 2008; Nichols and Vogt 2008; Devetak et al. 2004; Kanturski et al. 2016) as well as on other parts of the body (Catalá 1996). However, the antennae are the primary insect peripheral olfactory system (Chapman 1998; Keil 1999; de Bruyne and Baker 2008). In many cases, insect antennae have evolved into sophisticated shapes, such as feather- and club-shaped structures, in order to maximize the area that carries the odour detecting organs (Keil 1999).

According to their sensory modality, the sensilla of insects are classified into four main groups—olfactory, gustatory, mechanosensory and those that have hygro- and thermoreceptors. Numerous sensilla occur on antennae in the form of hairs, pegs, pits or cones. Depending on the cuticular structure, the sensilla are classified into different types (Slifer 1970; Altner and Prillinger 1980; Hallberg and Hansson 1999). The most common types are trichoid sensilla, long hairlike sensilla, basiconic sensilla (also hairlike but normally shorter and thicker than the trichoid sensilla), placoid sensilla, platelike and coeloconic sensilla (short peg-like structures that are situated in pits) (Hallberg and Hansson 1999; Shields 2010). Several studies have also shown a variety of sensilla in insects that are based on the presence of pores (Altner and Prillinger 1980; Zacharuk 1980; Shields 2010). Altner and Prillinger (1980) classified them into three main groups—no pores (aporous), terminal pores (uniporous) and wall pores (multiporous). Each group is allocated to a different function. Sensilla with no pores are typically mechanoreceptors (McIver 1975; Altner and Prillinger 1980). They can be also responsible for thermo- and hygroreception (Steinbrecht 1997; Hallberg et al. 2003). Sensilla that have pores are chemoreceptors. Uniporous sensilla are responsible for contact chemoreception or gustation, while multiporous sensilla are olfactory sensilla (Schneider and Steinbrecht 1968; Zacharuk 1980). The sensilla system of insects shows a remarkable morphological diversity. This diversity probably reflects selection pressures for high sensitivity, phylogenetic and/or developmental constraints and the physical environment in which the evolution took place, rather than adaptations designed to detect specific volatile chemicals (Hansson and Stensmyr 2011).

The majority of information regarding the antennal sensilla in Heteroptera is mainly based on hematophagous species of the family Reduviidae (Catalá 1997; Gracco and Catalá 2000; Guerenstein and Guerin 2001; Carbajal de la Fuente and Catalá 2002; Slu 1980). Antennal sensilla have also been studied in phytophagous families such as Miridae (Chinta et al. 1997), Alydidae (Rani and Madhavendra 2005; Ventura and Panizzi 2005), Lygaeidae (Rani and Madhavendra 2005), Pentatomidae (Rani and Madhavendra 1995; Brézot et al. 1997; Sinitsina and Chaika 1998; Ahmad et al. 2016) and Coreidae (Akent’eva 2008). Antennal sensilla in predatory gerromorphan species have not yet been identified. The knowledge of types and morphologies of sensilla contributes to a better understanding of the role that these structures play in the selection of a host.

In previous studies on Gerromorpha, the labial tip sensilla were investigated in detail (Brożek and Zettel 2014) and the sensilla (mechanosensilla) of some parts of the body surface (Andersen 1982). Hence, in the present study, we examined the external morphology of the antennal sensilla of the same taxon. Such an approach allows the exploration of a specific sensitivity profile in Gerromorpha, a taxa which occupies a different ecological niche compared to other groups of Heteroptera.

The aim of this study was therefore to investigate the diversity of antennal sensilla in Gerromorpha in order to (1) report on their disparity and (2) to compare it with what is already known is other Heteroptera taxa.

## Materials and methods

The morphology and distribution of the antennal sensilla of Gerromorpha were investigated in five of the eight families of Gerromorpha. Sexual dimorphism was not observed.

The species were not washed and dehydrated before the microscopic observation. Therefore, we assume that single openings (deep pore), are sensilla ampulaceae found on the surface of the antennae and not the apertures of tegumentary glands. Canals of tegumentary glands are usually covered by fluid, rendering them invisible, so the material should be cleaner before observation. Moreover, in other insect species, a canal of tegumentary glands is distinguished when surrounded by small, porous plates.

This study is based on the dry material of 21 species from 13 subfamilies. Species of the families Paraphrynoveliidae, Macroveliidae and Hermatobatidae were not included. All of the material was coated with a gold film and photographed using a Hitachi scanning electron microscope in the scanning microscopy laboratory of the Faculty of Biology and Environmental Protection of Silesian University in Katowice. The species that were examined were obtained from the Natural History Museum in Vienna.

*Examined species*

**Mesoveliidae: Mesoveliinae:** *Mesovelia furcata* Mulsant & Rey, 1852.

**Hebridae: Hebrinae:** *Hebrus philippinus* Zettel, 2006.

**Hydrometridae: Hydrometrinae:** *Hydrometra stagnorum* Linnaeus, 1758.

**Veliidae: Halovelinnae:** *Halovelia esakii* Andersen, 1989; *Strongylovelia philippinensis* Lansbury and Zettel, 1997.

**Microveliinae:** *Microvelia douglasi* Scott, 1874; *Pseudovelia pusilla* Hecher, 1997; *Neoalardus typicus* Distant, 1912.

**Rhagoveliinae:** *Rhagovelia kawakamii* Matsumura, 1913;

**Perittopinae:** *Perittopus asiaticus* Zettel, 2001;

**Veliinae:** *Velia caprai* Timanini, 1947; *Paravelia basalis* Spinola, 1837.

**Gerridae: Trepobatinae:** *Pseudohalobates inobonto* Polhemus & Polhemus 1996;

**Gerrinae:** *Gerris lacustris* Linnaeus, 1758; *Aquarius paludum* Fabricius, 1794;

**Eotrechinae:** *Amemboa javanica* Lundblad, 1933; *Amemboa cristata* Polhemus & Andersen, 1984;

*Amemboa brevifasciata* Miyamoto, 1967; *Onychotrechus esakii* Andersen, 1980;

**Ptilomerinae:** *Rheumatogonus luzonicus* Kirkaldy, 1909;

**Halobatinae:** *Metrocoris nigrofascioides* Chen & Nieser, 1993.

## Results

### Sensilla types in the examined species

**Fig. 1:**
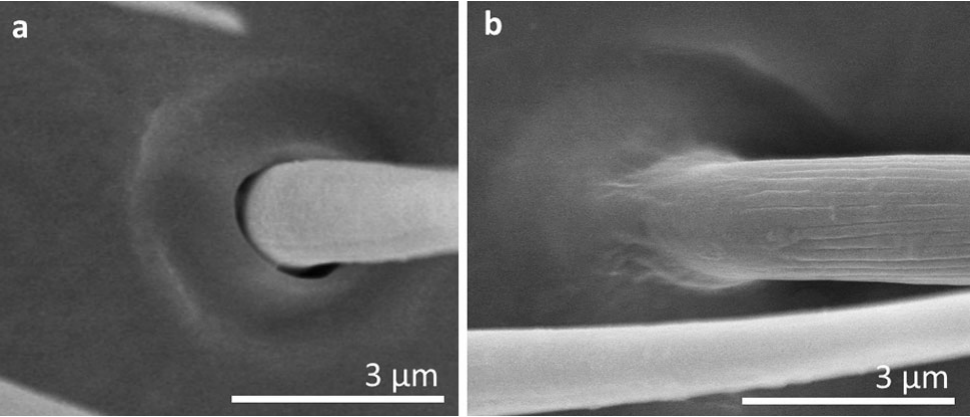
Types of sockets: **a** flexible socket; **b** inflexible socket

Based on size, shape, presence or absence of pores, their distribution and cuticular attachment (flexible or inflexible socket), nine types (I–IX) of antennal sensilla were identified in the adult specimens. They were classified as: sensilla trichoidea (ST), sensilla chaetica (SCh), sensilla leaflike (SL), sensilla campaniforme (SCa), sensilla coeloconica (SCo), sensilla ampullacea (SA), sensilla basiconica (SB) and sensilla placoidea (SP). In addition, we distinguished a specific morphological structure in one of the types of sensilla. It is a funnel-shaped pit with a short sensory cone that has cuticular folds. Generally, the sensilla arise from cuticular sockets. The flexible sockets have a thin cuticular membrane, which is continuous with the general body cuticle and the hair (Fig. 1a). It provides greater mobility at the base of the sensilla. Some sensilla can also arise from a cuticle without a membrane with a specialised socket region (inflexible socket) (Fig. 1b). The characteristic morphological features of the antennal sensilla and their classification are based on the papers of McIver 1975, Altner and Prillinger 1980, Hallberg and Hansson 1999, Shields 2010.

**Fig. 2:**
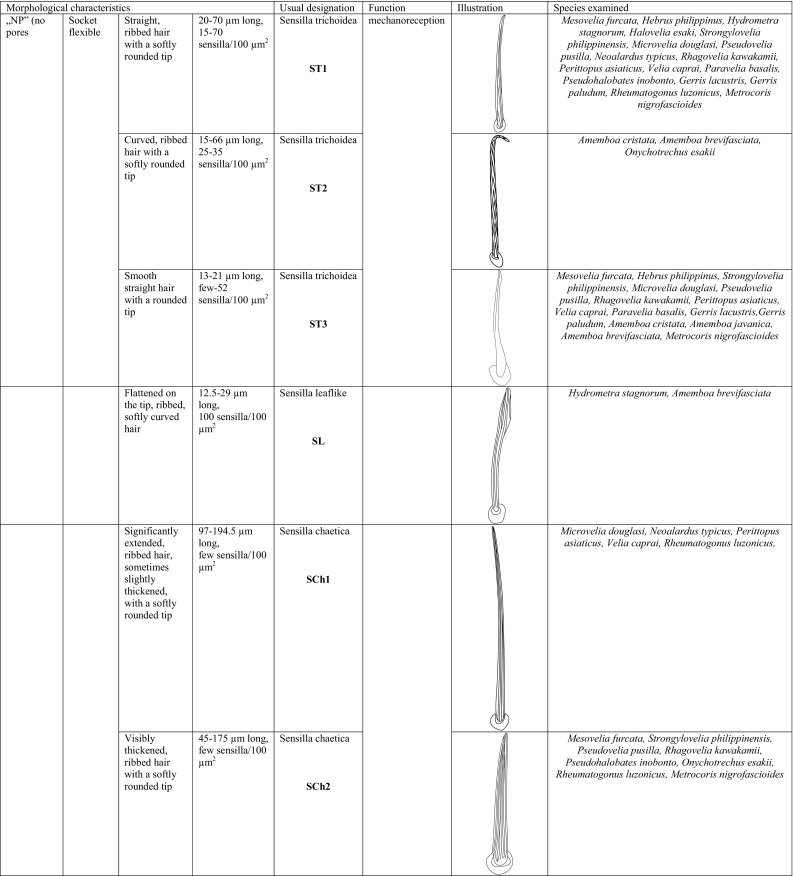

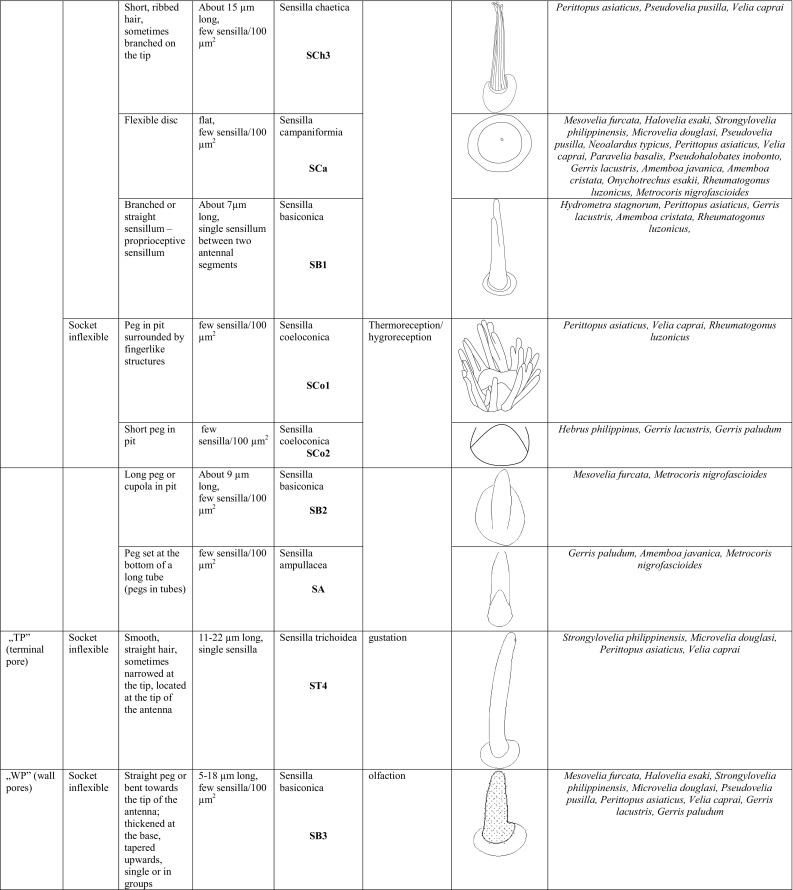

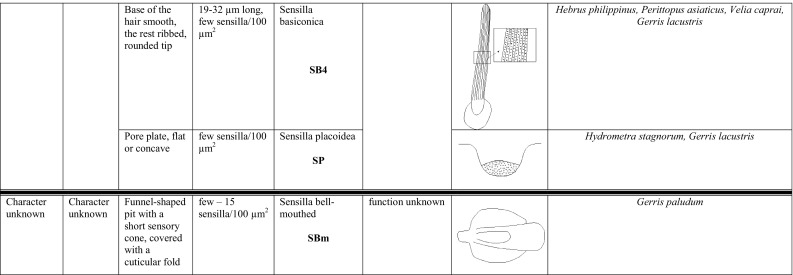
Diagram of sensilla types and the species tested

**Fig. 3:**
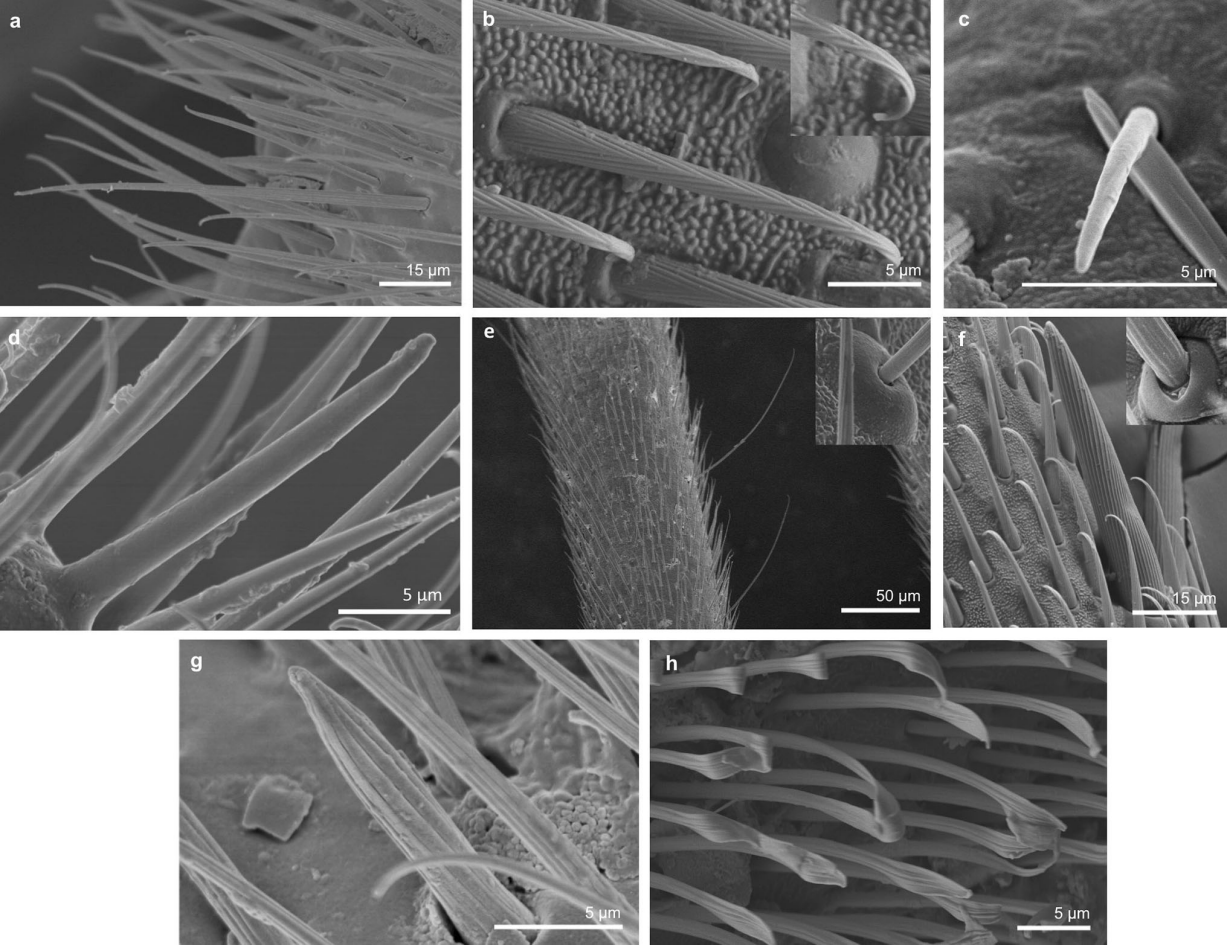
Main sensilla types: **a** sensilla trichoidea 1, **b** sensilla trichoidea 2, **c** sensilla trichoidea 3, **d** sensilla trichoidea 4, **e** sensilla chaetica 1, **f** sensilla chaetica 2, **g** sensilla chaetica 3, **h** sensilla leaflike

**Fig. 4:**
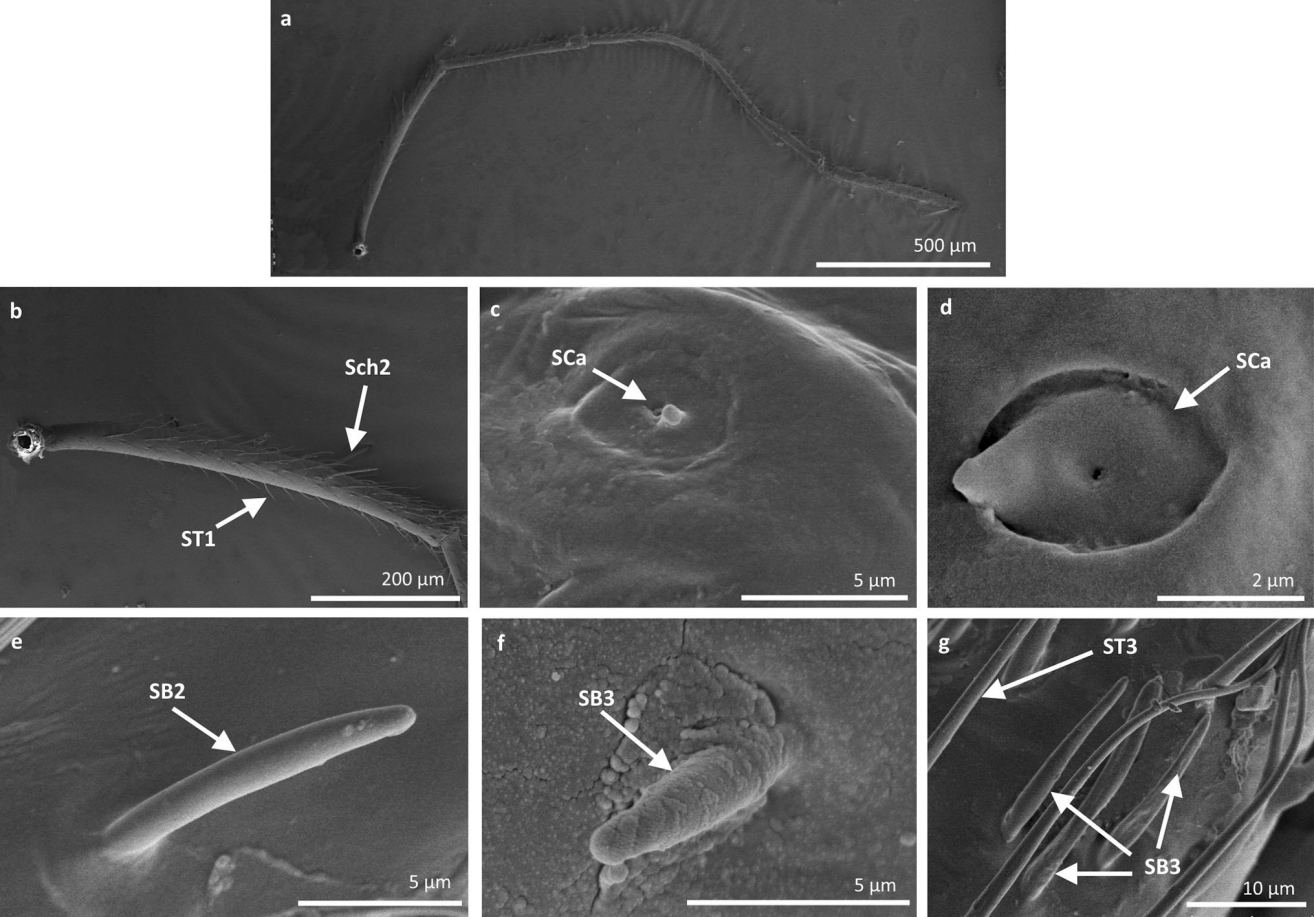
Sensilla types in Mesoveliidae: **a–g** *Mesovelia furcata*; *ST1* sensilla trichoidea 1, *SC2* sensilla chaetica 2, *SCa* sensilla campaniformia, *SB2* sensilla basiconica 2, *SB3* sensilla basiconica 3, *ST3* sensilla trichoidea 3

**Fig. 5:**
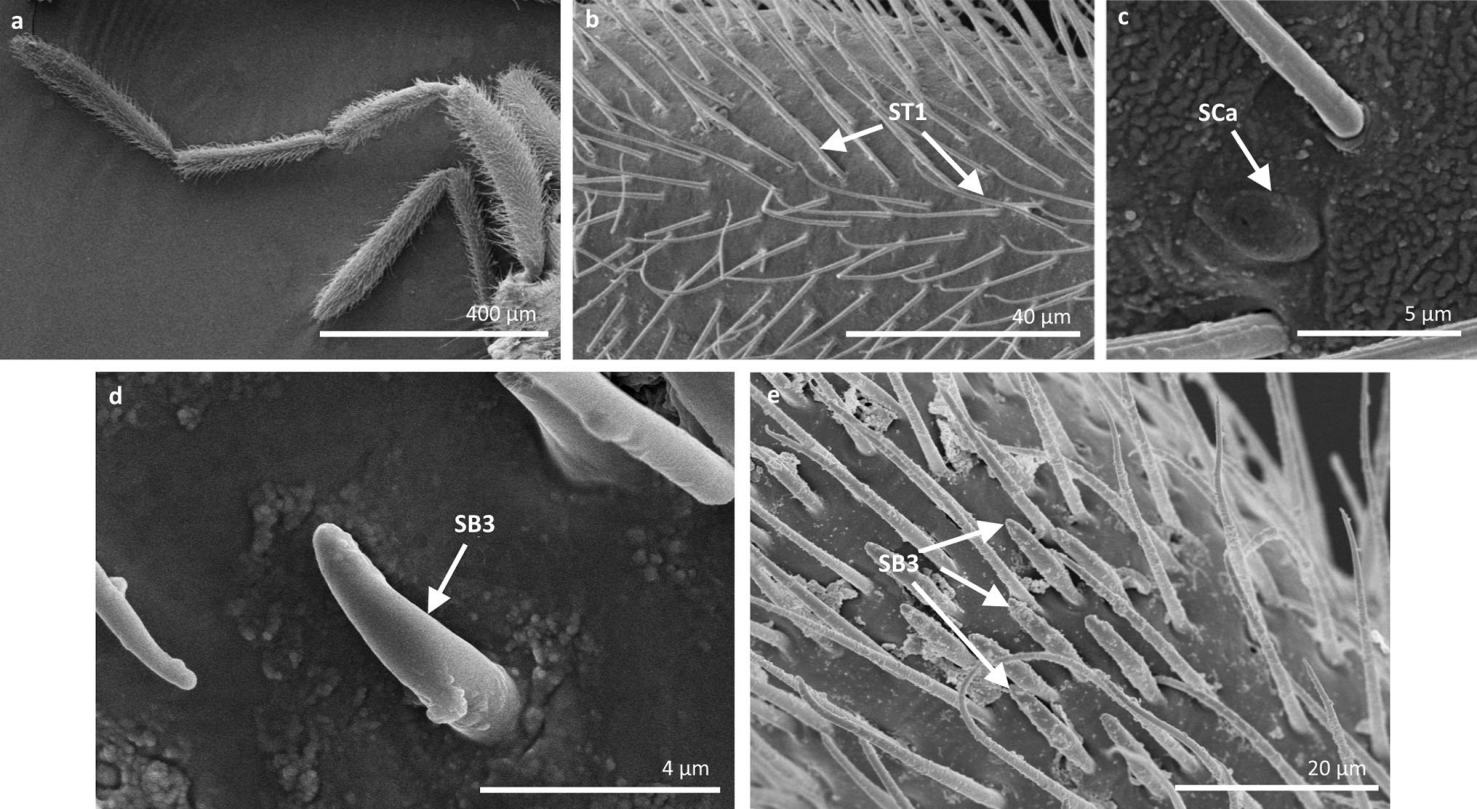
Sensilla types in Veliidae: **a–e** *Halovelia esakii*; *ST1* sensilla trichoidea 1, *SCa* sensilla campaniformia, *SB3* sensilla basiconica 3

**Fig. 6:**
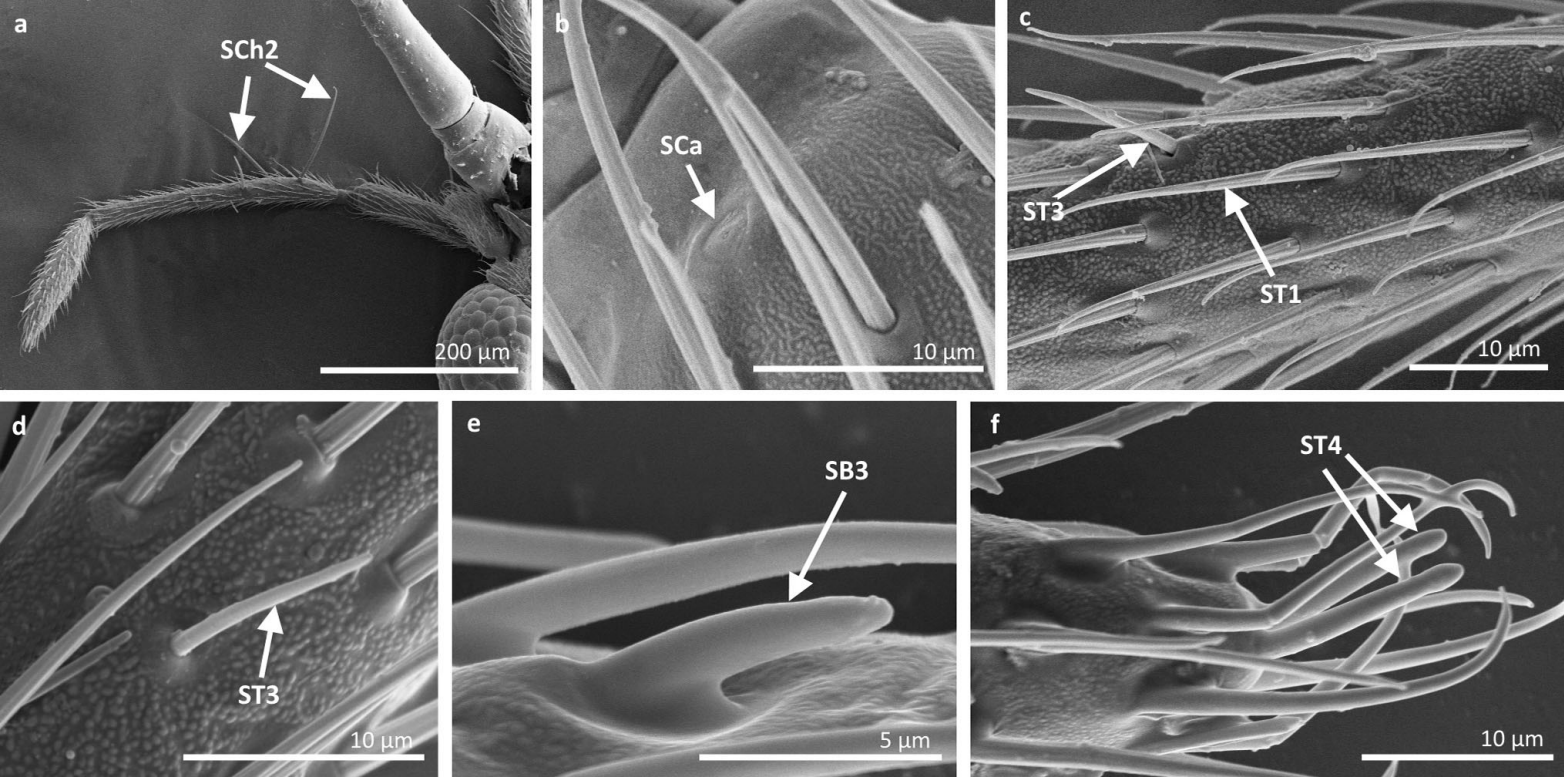
Sensilla types in Veliidae: **a–f** *Strongylovelia philippinensis*; *SCh2* sensilla chaetica 2, *SCa* sensilla campaniformia, *ST3* sensilla trichoidea 3, *ST1* sensilla trichoidea 1, *SB3* sensilla basiconica 3, *ST4* sensilla trichoidea 4

**Fig. 7:**
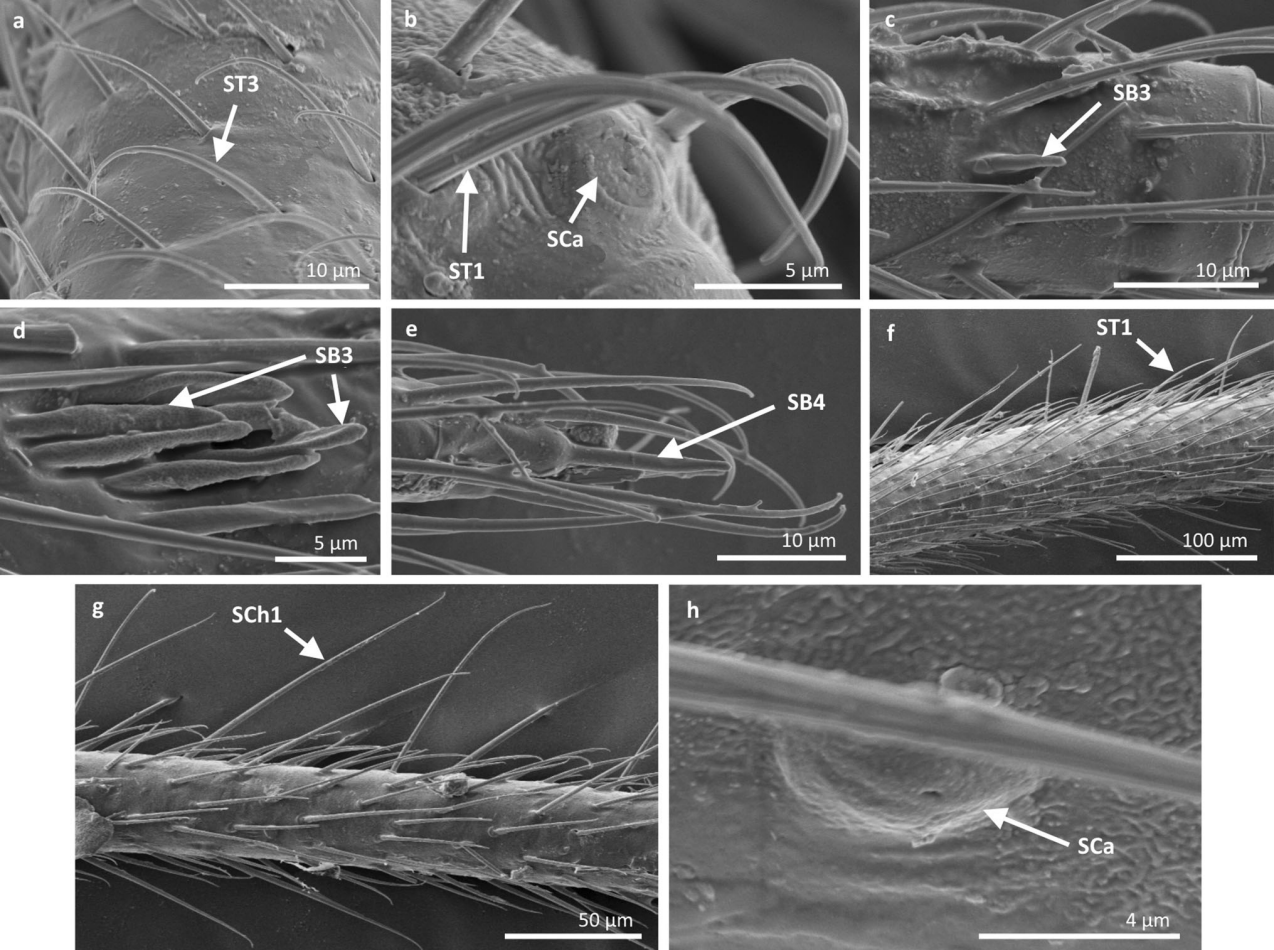
Sensilla types in Veliidae: **a–e** *Microvelia douglasi*, **f–h** *Neoalardus typicus*; *ST3* sensilla trichoidea 3, *ST1* sensilla trichoidea 1, *SCa* sensilla campaniformia, *SB3* sensilla basiconica 3, *ST4* sensilla trichoidea 4, *SCh1* sensilla chaetica 1

**Fig. 8:**
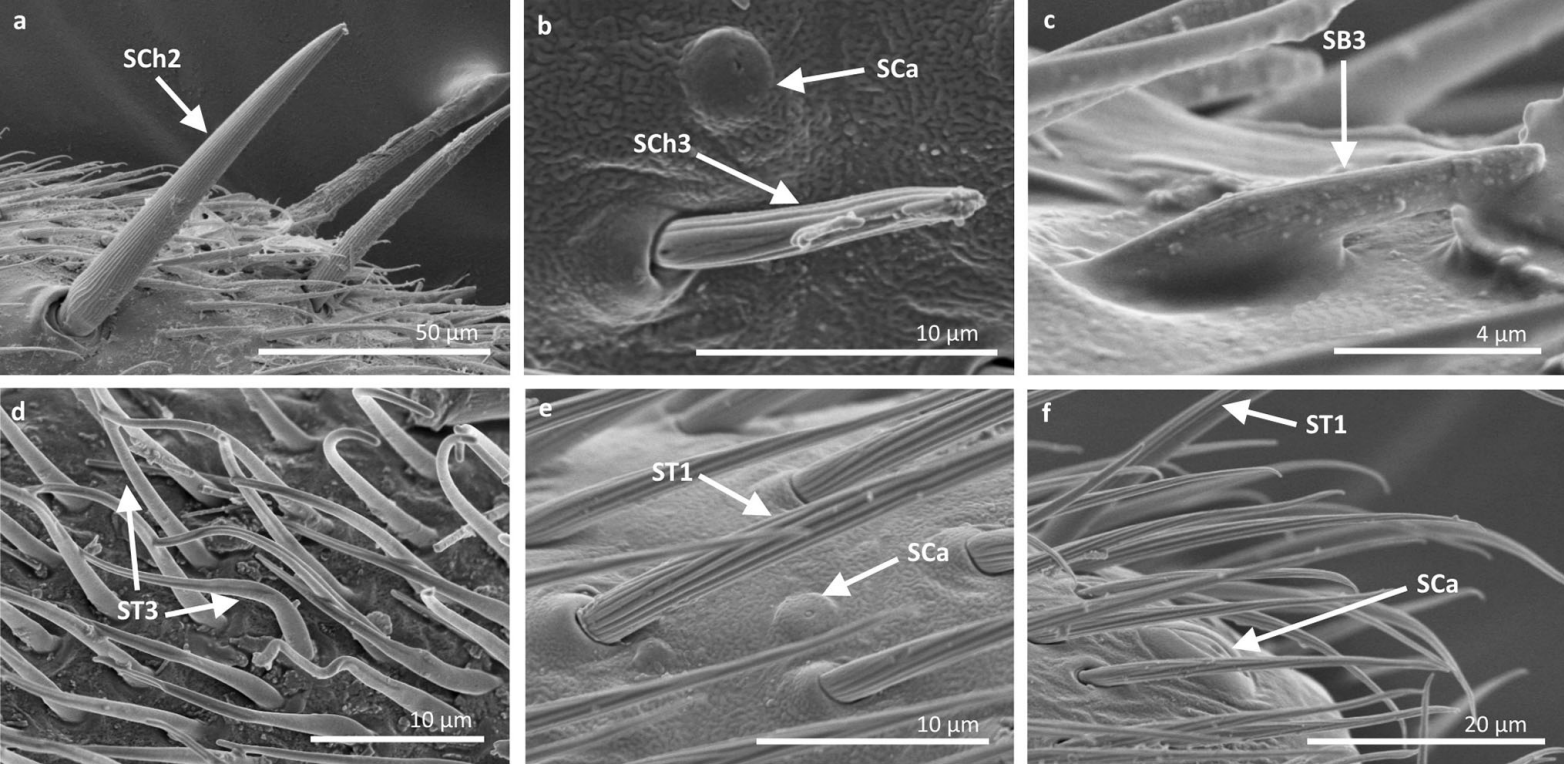
Sensilla types in Veliidae: **a–f** *Pseudovelia pusilla*; *SCh2* sensilla chaetica 2, *SCh3* sensilla chaetica 3, *SCa* sensilla campaniformia, *SB3* sensilla basiconica 3, *ST3* sensilla trichoidea 3, *ST1* sensilla trichoidea 1

**Fig. 9:**
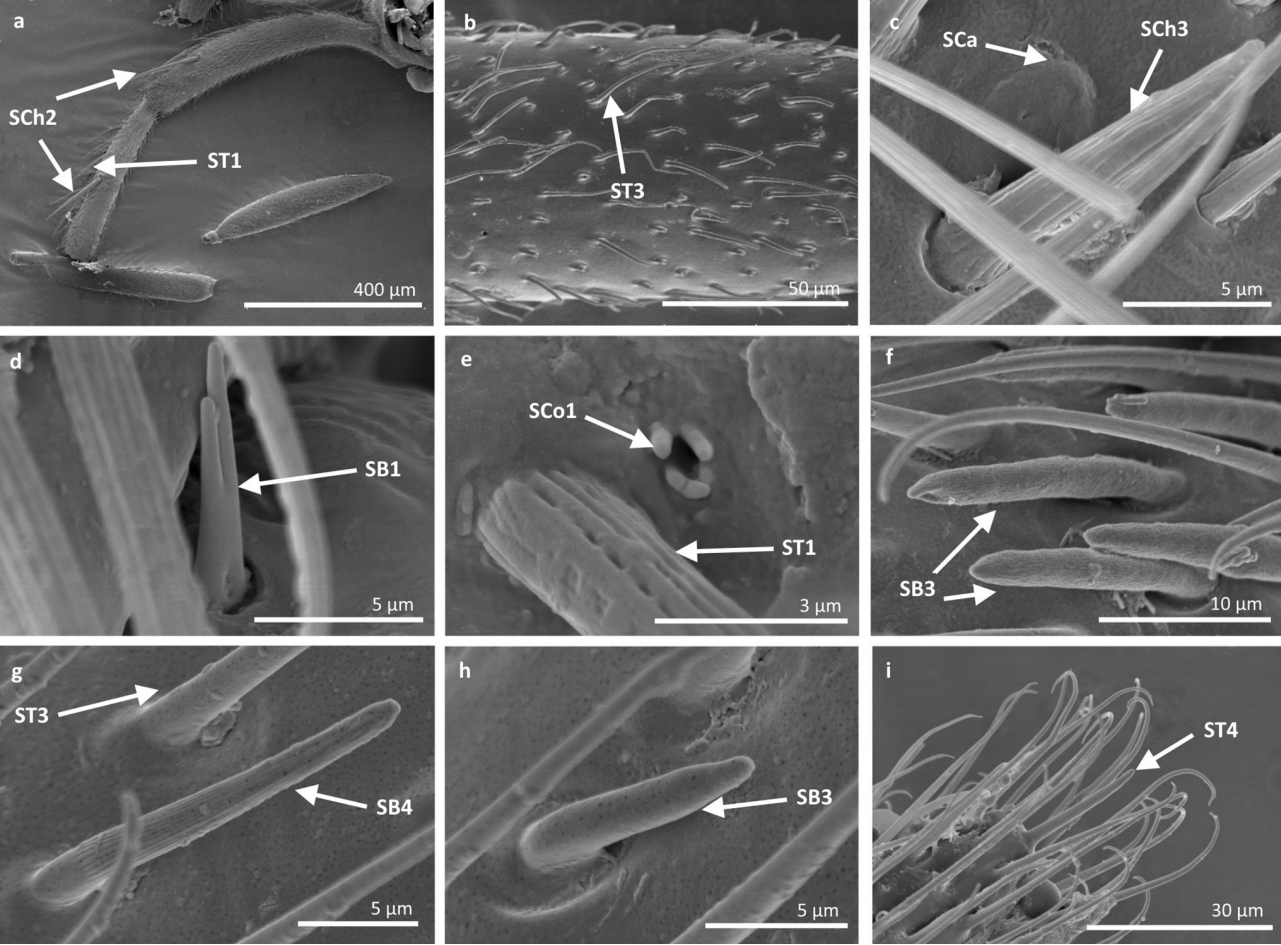
Sensilla types in Veliidae: **a**, **b** *Rhagovelia kawakamii*, **c–i** *Perittopus asiaticus*; *SCh2* sensilla chaetica 2, *ST1* sensilla trichoidea 1, *ST3* sensilla trichoidea 3, *SCa* sensilla campaniformia, *SCh3* sensilla chaetica 3, *SB1* sensilla basiconica 1, *SCo1* sensilla coeloconica 1, *SB3* sensilla basiconica 3, *SB4* sensilla basiconica 4, *ST4* sensilla trichoidea 4

**Fig. 10:**
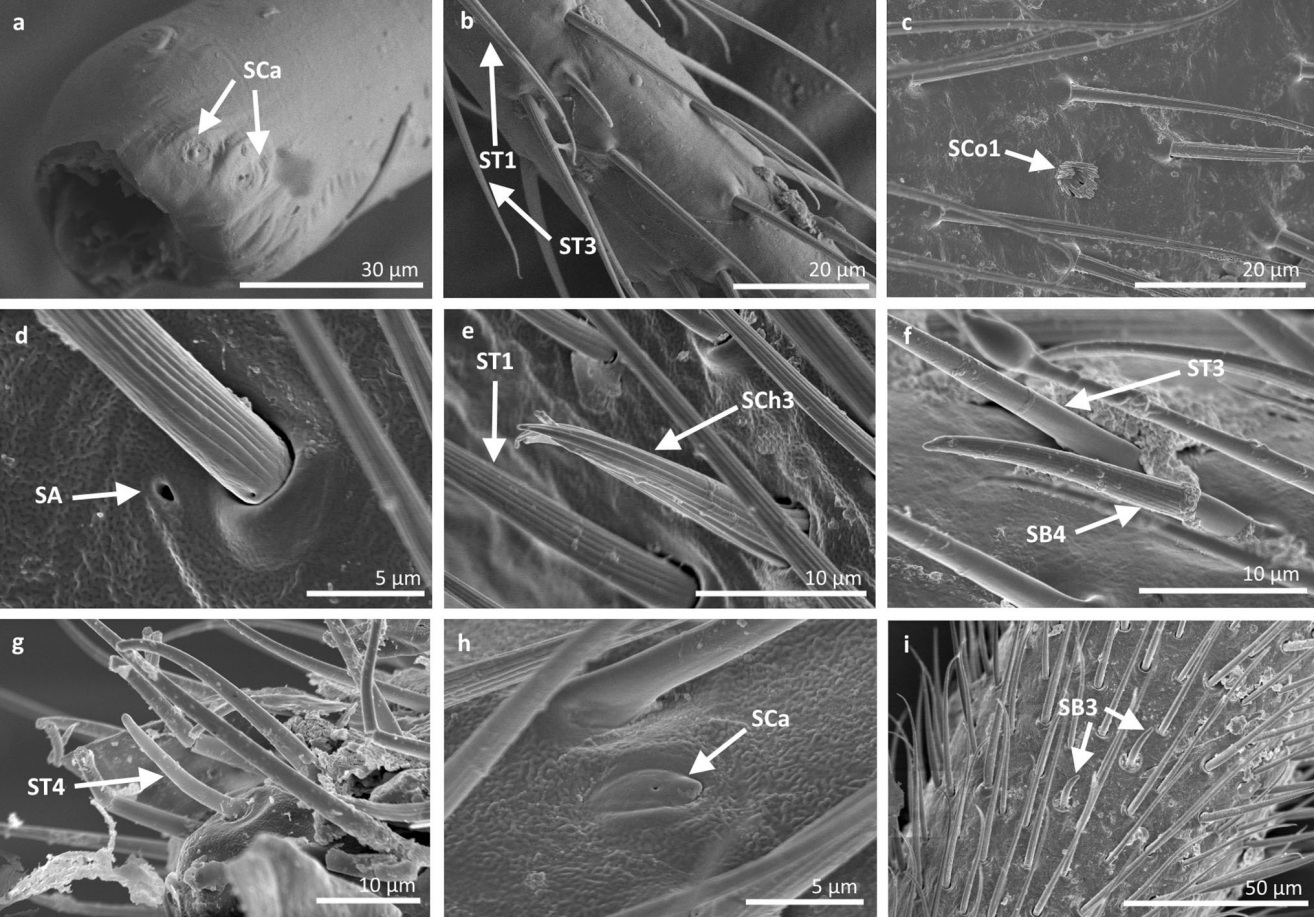
Sensilla types in Veliidae: **a**, **b** *Paravelia basalis*, **c–i** *Velia caprai*; *SCa* sensilla campaniformia, *ST1* sensilla trichoidea 1, *ST3* sensilla trichoidea 3, *SCo1* sensilla coeloconica 1, *SA* sensilla ampullacea, *SCh3* sensilla chaetica 3, *ST4* sensilla trichoidea 4, *SB3* sensilla basiconica 3

**Fig. 11:**
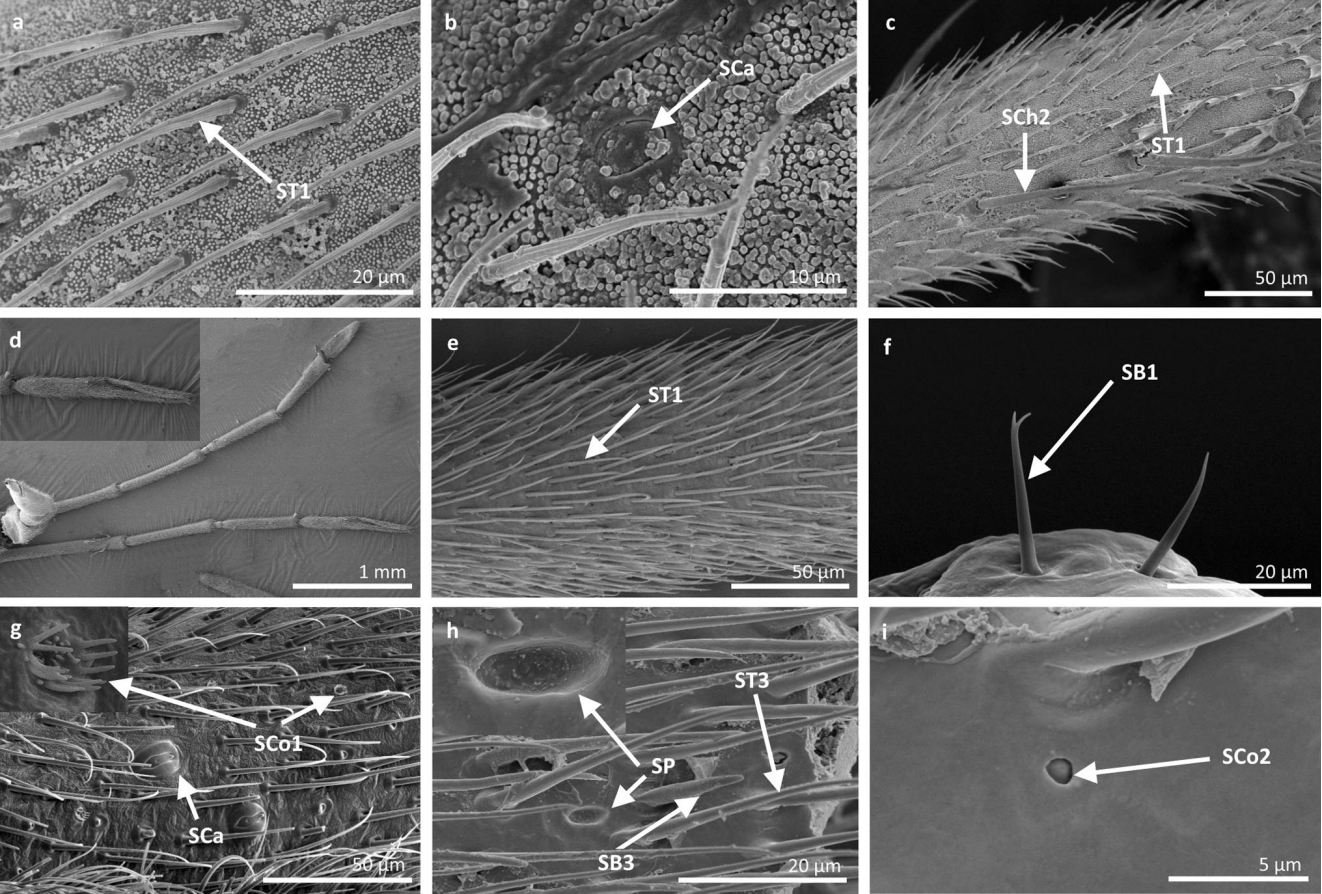
Sensilla types in Gerridae: **a–c** *Pseudohalobates inobonto*, **d–i** *Gerris lacustris*; *ST1* sensilla trichoidea 1, *SCa* sensilla campaniformia, *SCh2* sensilla chaetica 2, *SB1* sensilla basiconica 1, *SCo1* sensilla coeloconica 1, *SP* sensilla placoidea, *ST3* sensilla trichoidea 3, *SB3* sensilla basiconica 3, *SCo2* sensilla coeloconica 2
  2. *Sensilla trichoidea 2 (ST2)* These are 15–66 µm long and aporous, the sensilla are ribbed, curved at the tip and embedded in flexible sockets (Figs. 2, 3b). These sensilla are densely and uniformly distributed on the first and third antennomers and were only observed in the subfamily Eotrechinae (Gerridae) (Fig. 12a, h). There were no other types of sensilla on these antennomers.

I. *Sensilla trichoidea (ST)* These are flexible hairlike sensilla that vary in length (Fig. 2). The stem of the sensilla may be ribbed or smooth and usually tapers from the base to the tip. ST arises from flexible or inflexible cuticular sockets. These sensilla were observed on every antennomer in species that were analysed. Four subtypes of sensilla trichoidea were distinguished:

  1. *Sensilla trichoidea 1 (ST1)* These sensilla are 20–70 µm long, aporous, ribbed, with a slightly rounded tip and flexible sockets (Figs. 2, 3a). These sensilla are numerous and are densely and uniformly distributed on the antennomers. They are common and usually occur on the first and third or all of the antennomers in the representatives of the studied families (Figs. 4b, 5b, 6c, 7b, f, 8e, f, 9a, e, 10b, e, 11a, c, e).

**Fig. 12:**
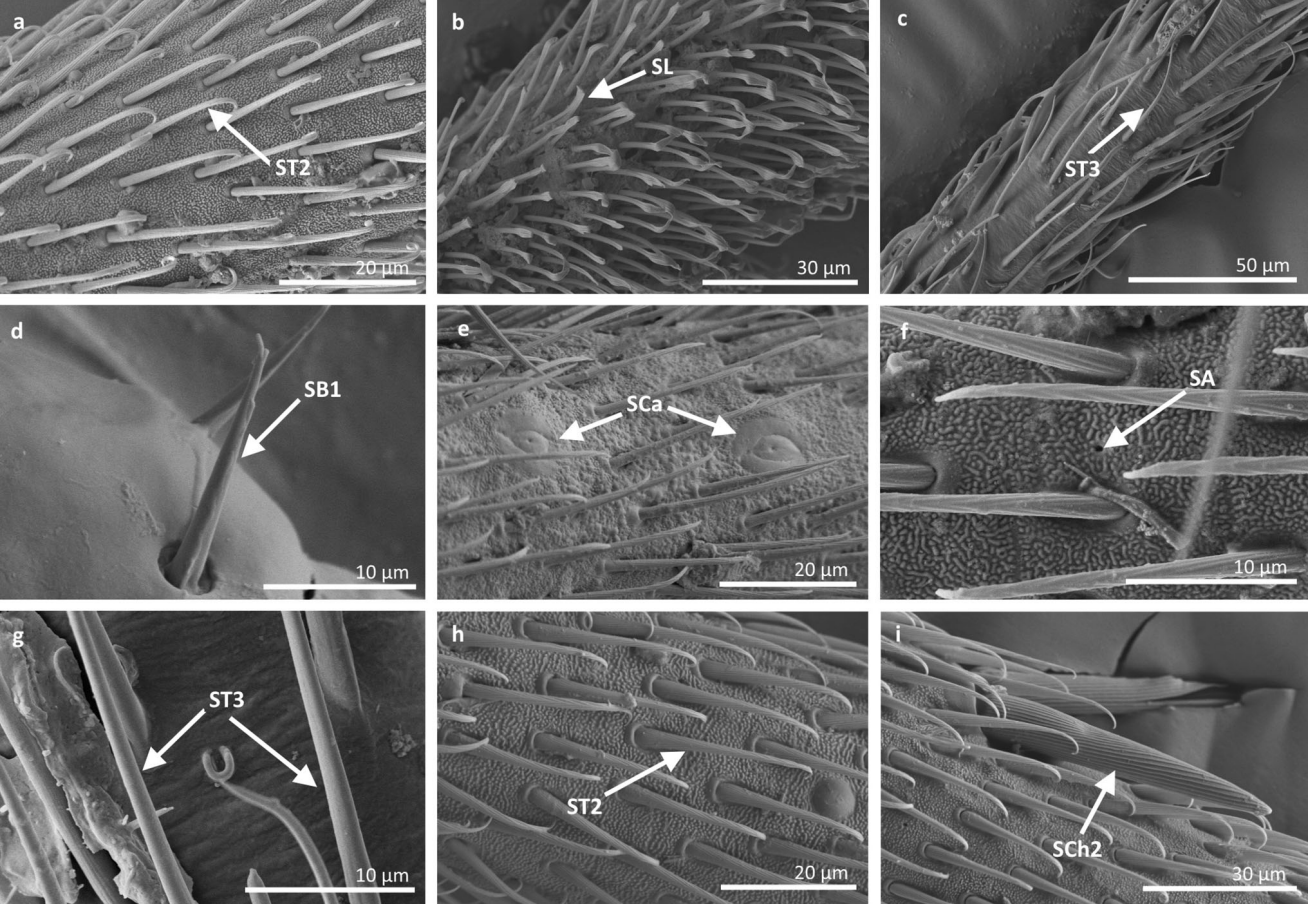
Sensilla types in Gerridae: **a–c** *Amemboa brevifasciata*, **d**, **e** *Amemboa cristata*, **f**, **g** *Amemboa javanica*, **h**, **i** Onychotrechus esakii; ST2 sensilla trichoidea 2, SL sensilla leaflike, ST3 sensilla trichoidea 3, *SB1* sensilla basiconica 1, *SCa* sensilla campaniformia, *SA* sensilla ampullacea, *SCh2* sensilla chaetica 2
  3. *Sensilla trichoidea 3 (ST3)* These sensilla are 13–21 µm long. They are aporous, straight, with a smooth surface, a rounded tip and flexible sockets (Figs. 2, 3c). They usually occur on the third and fourth antennomers. These sensilla are numerous and are quite densely distributed in most species such as Mesoveliidae (Fig. 4g), Hebridae (Fig. 13b, c), Veliidae (Figs. 6c, d, 7a, 8d, 9b, g, 10b, f) and Gerridae (Figs. 11h, 14c, 12c, g, 15b) but not on *Strongylovelia philippinensis* (Veliidae), in which a single such sensillum was observed on each antennomers (Fig. 6c, d).

**Fig. 13:**
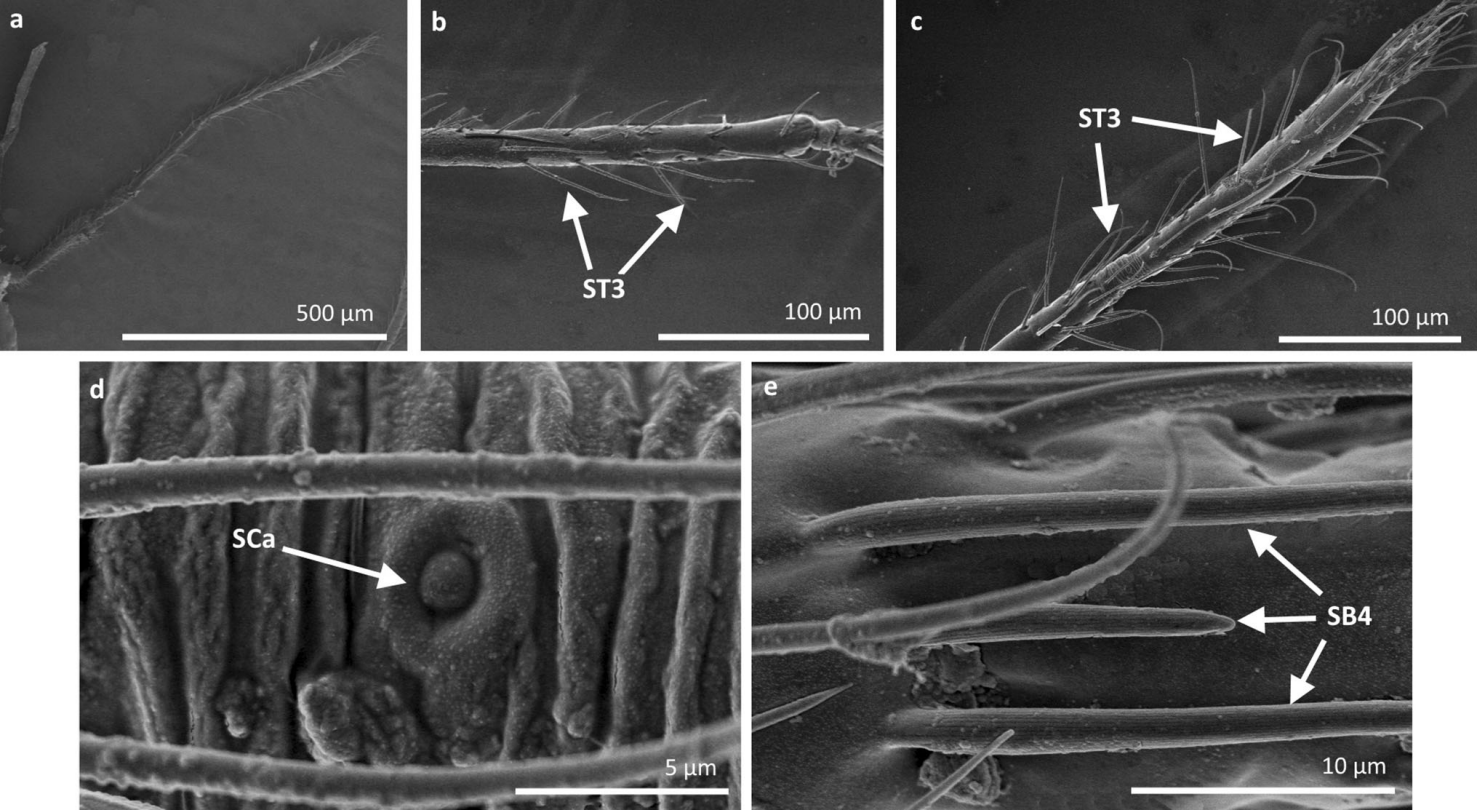
Sensilla types in Hebridae: **a–e** *Hebrus philippinus*; *ST3* sensilla trichoidea 3, *SCa* sensilla campaniformia, *SB4* sensilla basiconica 4

**Fig. 14:**
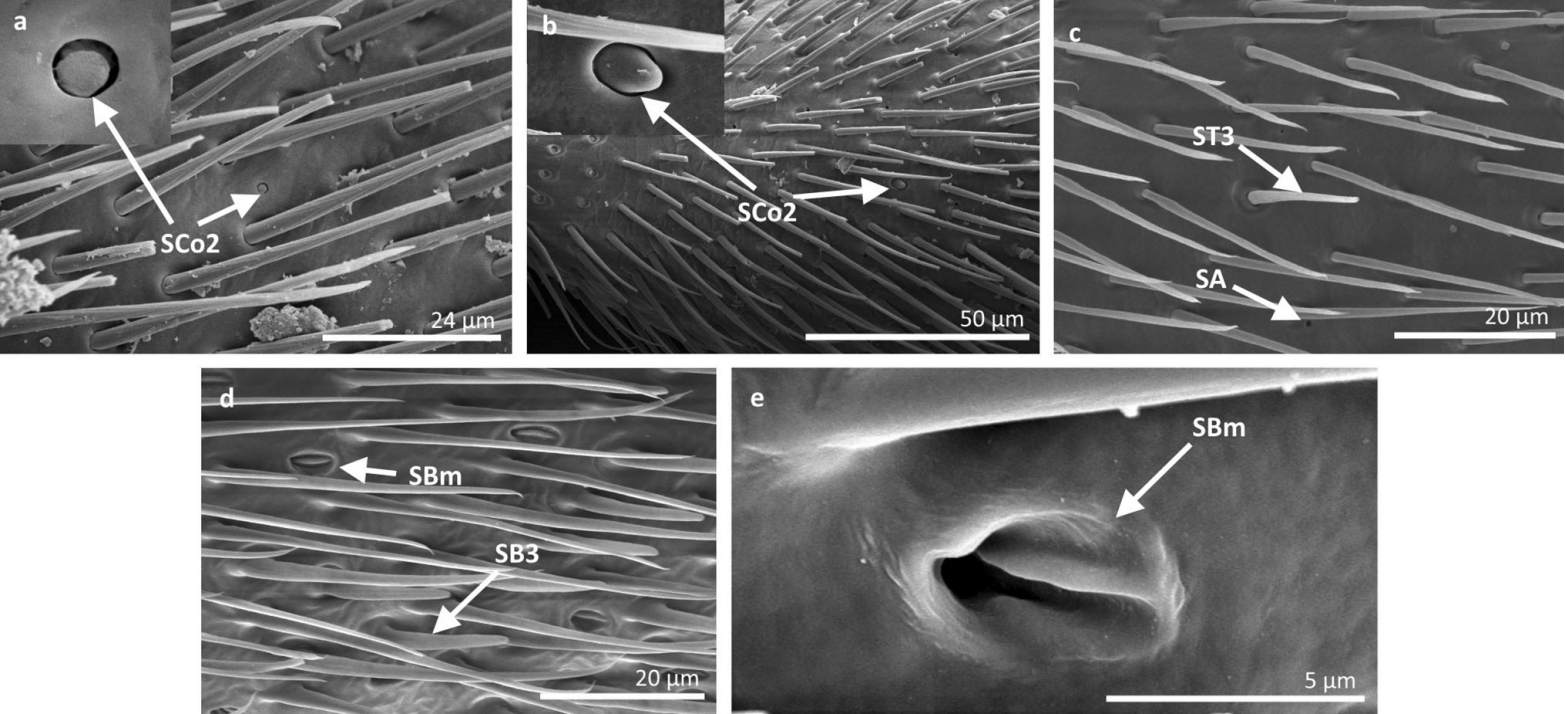
Sensilla types in Gerridae: **a–e** *Aquarius paludum*; *SCo2* sensilla coeloconica 2, *ST3* sensilla trichoidea 3, *SA* sensilla ampullacea, *SBm* sensilla bell-mouthed, *SB3* sensilla basiconica 3

**Fig. 15:**
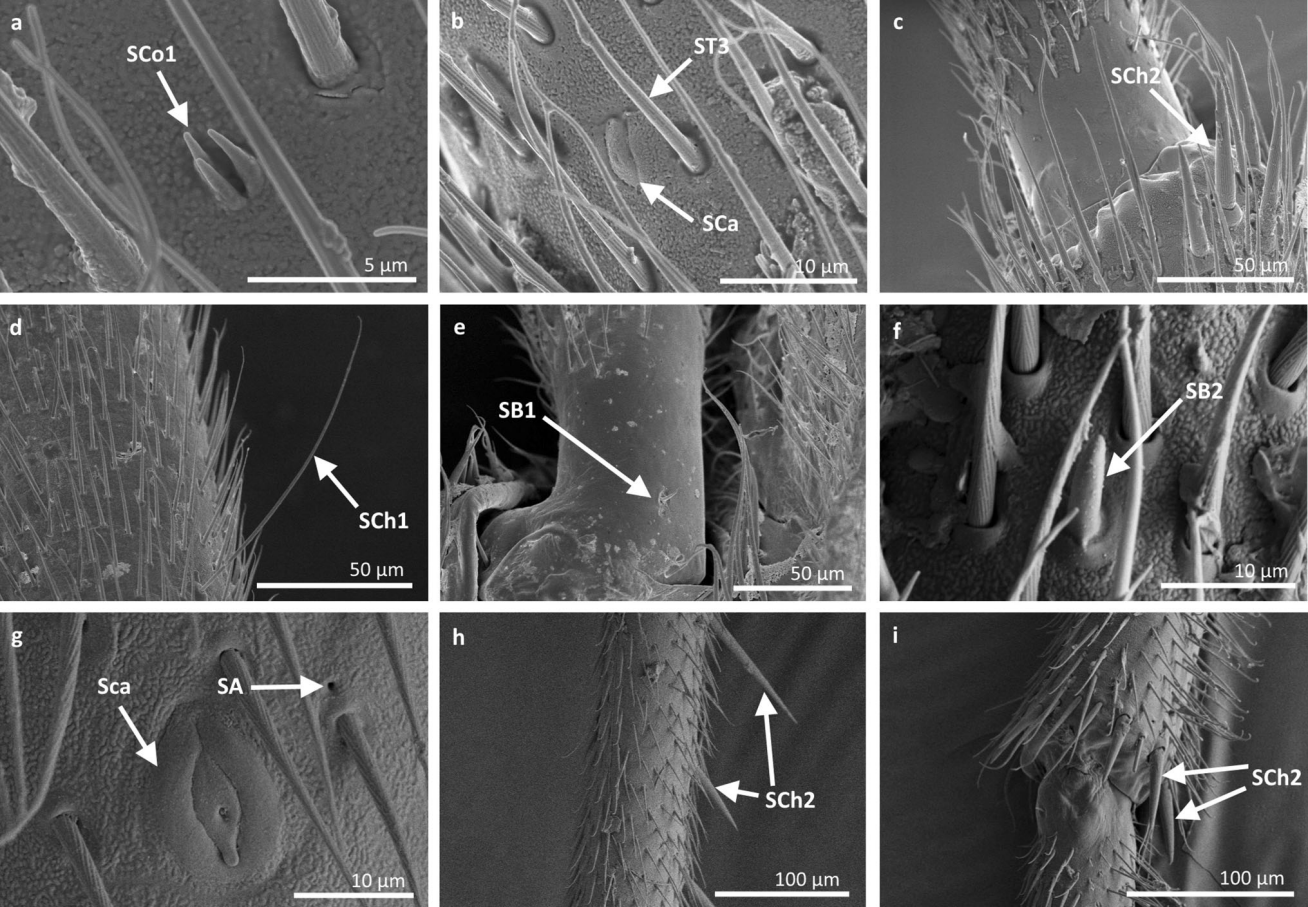
Sensilla types in Gerridae: **a–e** *Rheumatogonus luzonicus*, **f**–**i** *Metrocoris nigrofascioides*; *SCo1* sensilla coeloconica 1, *ST3* sensilla trichoidea 3, *SCa* sensilla campaniformia, *SCh2* sensilla chaetica 2, *SCh1* sensilla chaetica 1, *SB1* sensilla basiconica 1, *SB2* sensilla basiconica 2, *SA* sensilla ampullacea

**Fig. 16:**
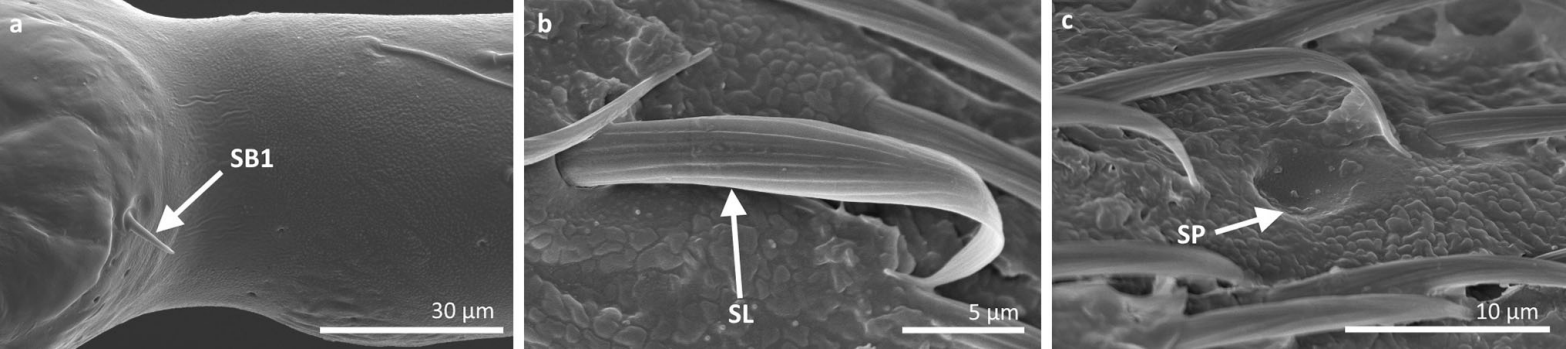
Sensilla types in Hydrometridae: **a–c** *Hydrometra stagnorum*; *SB1* sensilla basiconica 1, *SL* sensilla leaflike, *SP* sensilla placoidea
  4. *Sensilla trichoidea 4 (ST4)* These are 11–22-µm-long sensilla. Generally, they are straight with a smooth surface, narrowed at the tip and embedded in inflexible sockets (Figs. 2, 3d). A terminal pore was observed on the tip of this type of sensillum. This sensillum occurs at the tip of the antenna singularly, or with a few other STtp sensilla. This type of sensilla was only found in the family Veliidae (Figs. 6f, 7c, 9i, 10g).
II. *Sensilla chaetica* (*SCh*) These are stiff hairlike structures that are long and straight and thicker than sensilla trichoidea. All of their stems are ribbed and arise from a cuticle with a flexible socket. Three subtypes were differentiated:
  1. *Sensilla chaetica 1 (SCh1)* These are ribbed, elongated sensilla (97–195 µm long), aporous, with a rounded tip and a flexible socket (Figs. 2, 3e). They are quite densely spread on all of the antennomers or few individual chaetica occur on one of them. This type of sensillum was found in the family Veliidae (Fig. 7g) and Gerridae (Fig. 15d).
  2. *Sensilla chaetica 2 (SCh2)* These are ribbed, 45–175 µm long sensilla, aporous, visibly thicker than other sensilla chaetica, with a rounded tip and a flexible socket (Figs. 2, 3f). A few sensilla usually occur on the first or second antennomer or they are more numerous and surround the end of the first antennomer. This type of sensillum occurs in the families Mesoveliidae (Fig. 4b), Veliidae (Figs. 6a, 8a, 9a) and Gerridae (Figs. 11c, 12i, 15c, h, i).
  3. *Sensilla chaetica 3 (SCh3)* These sensilla are ribbed, short (15 µm long), aporous, straight and are slightly branched at the tip in some species (Figs. 2, 3g). These sensilla have flexible sockets and are individually distributed on the first, second and third antennomers. This type of sensillum was characteristic of the family Velii dae (Figs. 8b, 9c, 10e).
III. *Sensilla leaflike (SL)* These are ribbed, 12–30-µm-long sensilla. Their stems are straight and the tips of the sensilla are slightly curved, pointed or flattened (Figs. 2, 3h). These sensilla are embedded in flexible sockets. This type of sensillum was found on the first and second antennomers in two species—*Hydrometra stagnorum* (Fig. 16b) and *Amemboa brevifasciata* (Fig. 12b). They are densely distributed (Fig. 2) and there were no other sensilla on the antennomers.
IV. *Sensilla campaniformia (SCa)* These are flat, oval-shaped discs with a single pore molting observed on their surface (Figs. 2, 17a). They are sparsely distributed on different segments of the antenna and have flexible sockets. This type of sensillum occurs in the families Veliidae (Veliinae and Perittopinae) (Figs. 9e, 10c) and Gerridae (Ptilomerinae) (Figs. 11g, 15a).

**Fig. 17:**
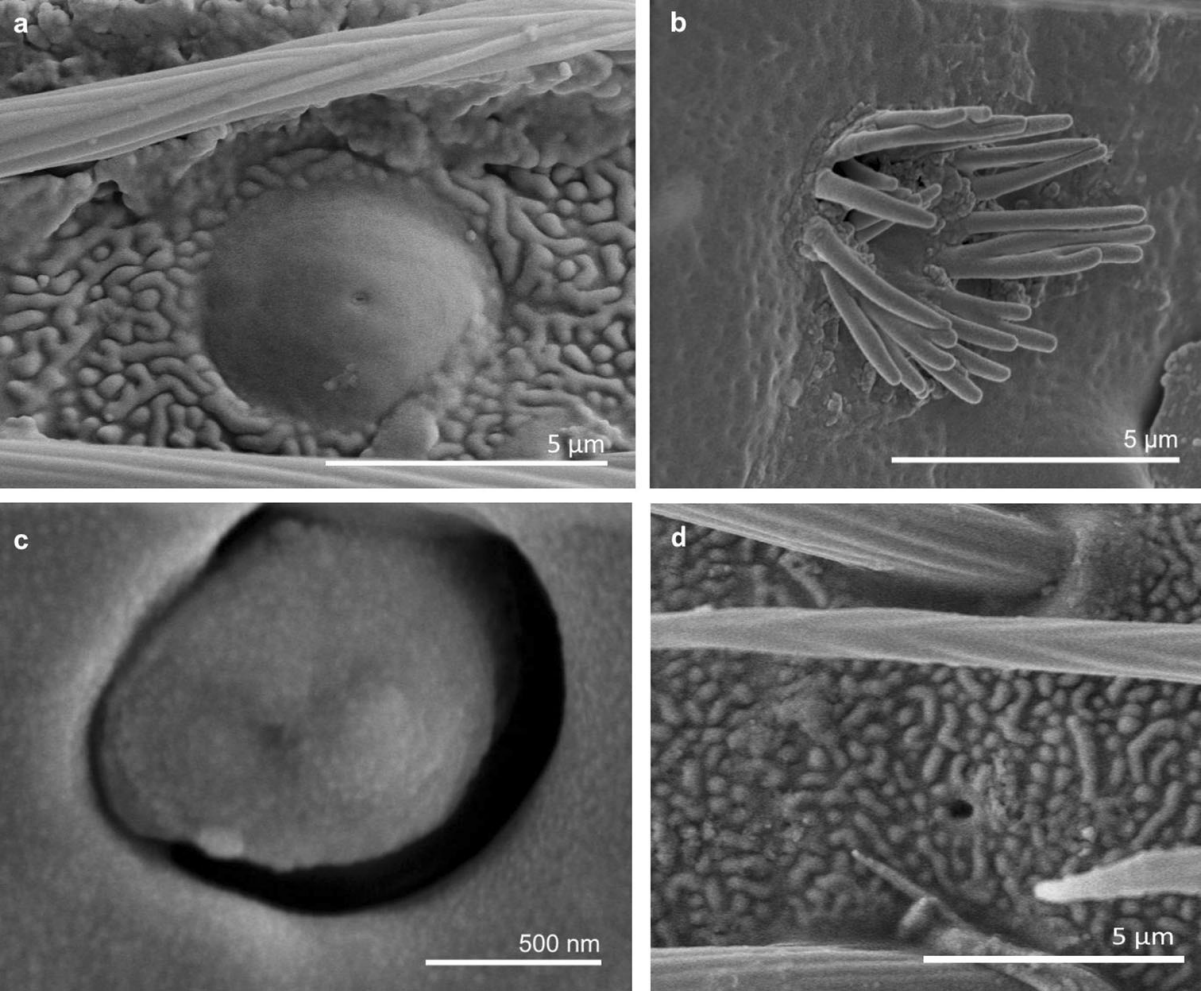
Main sensilla types: **a** sensilla campaniformia, **b** sensilla coeloconica 1, **c** sensilla coeloconica 2, **d** sensilla ampullacea
V. *Sensilla coeloconica (SCo)* These are short pegs in the pit sensilla that have a smooth surface and that are concealed in the cavity of a cuticle. They may be hidden and almost invisible on the surface or they may be discernible. They are embedded in inflexible sockets.
  1. *Sensilla coeloconica 1 (SCo1)* These are individual sensilla that are concealed in the cavity and are surrounded by various number of fingerlike structures (Figs. 2, 17b). A few sensilla were observed on the first and second antennomers. This type of sensillum occurs in the families Veliidae (Figs. 9e, 10c) and Gerridae (Figs. 11g, 15a).
  2. *Sensilla coeloconica 2 (SCo2)* These are individual sensilla that slightly grow over the cavity (Figs. 2, 17c). They are sparsely distributed on the surface of the antenna. They were found in the subfamilies Mesoveliinae (Mesoveliidae), Hebrinae (Hebridae) (Fig. 13d) and Gerrinae (Gerridae) (Figs. 11i, 14a, b).
VI. *Sensilla ampullacea (SA)* These sensilla are pegs set at the bottom of a tube internally but appear as small round openings on the cuticular surface externally (Figs. 2, 17d). They were observed in the family Gerridae (Figs. 14c, 12f, 15g).
VII. *Sensilla basiconica (SB)* These sensilla are cones that arise from flexible or inflexible sockets. Their surface may be porous or aporous. Four subtypes of sensilla basiconica were identified:
  1. *Sensilla basiconica 1 (SB1)* These are straight or branched cones that are about 7 µm long with a smooth surface and a flexible socket (Figs. 2, 18a). They occur as individual structures between two antennal segments and are usually found between the first and second segment. This type of sensillum was found in the families Hydrometridae (Fig. 16a), Veliidae (Fig. 9d) and Gerridae (Figs. 11f, 12d, 15e). They have a flexible socket.

**Fig. 18:**
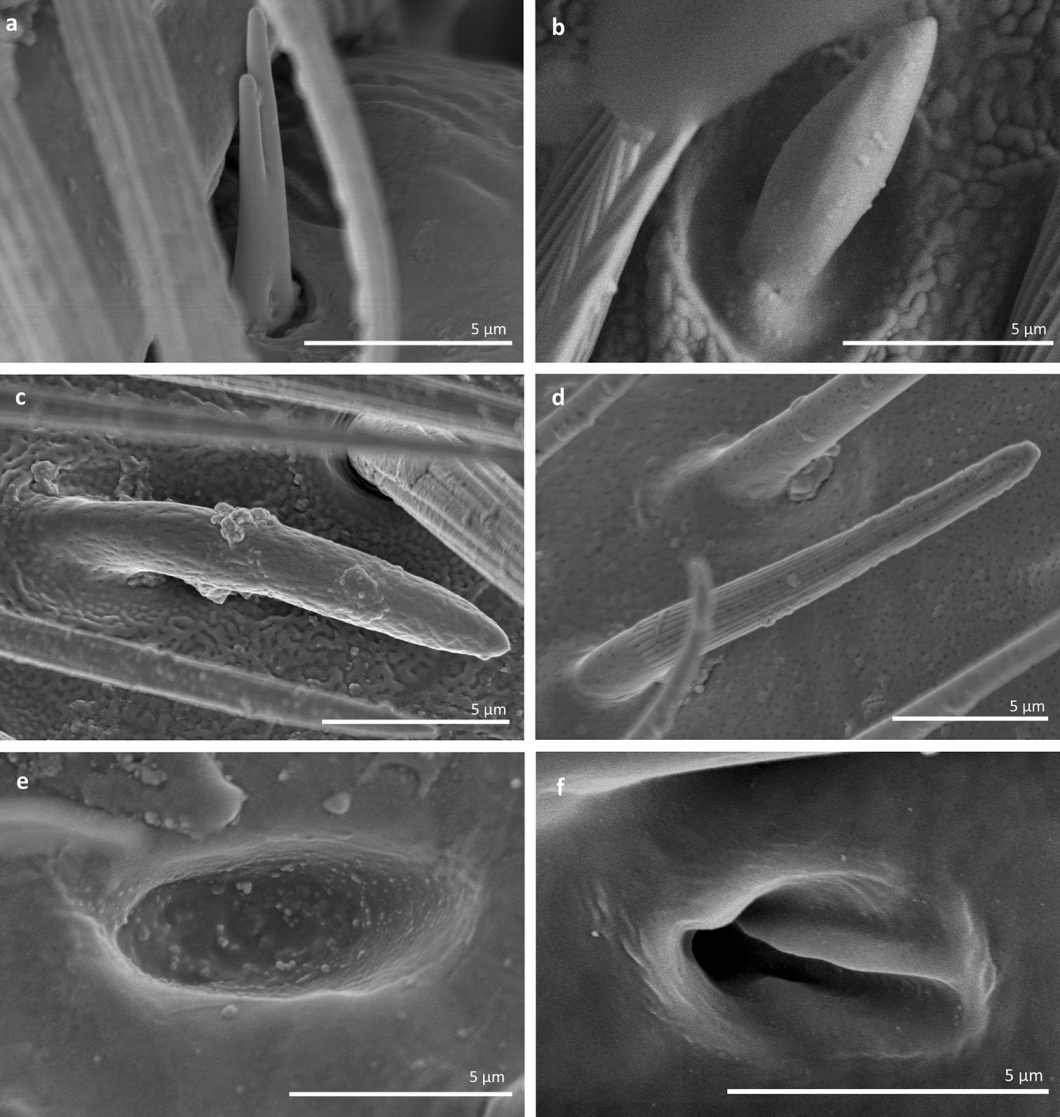
Main sensilla types: **a** sensilla basiconica 1, **b** sensilla basiconica 2, **c** sensilla basiconica 3, **d** sensilla basiconica 4, **e** sensilla placoidea, **f** sensilla bell-mouthed
  2. *Sensilla basiconica 2 (SB2)* These are single peg sensilla that are about 9 µm long with no pores and inflexible socket (Figs. 2, 18b). They are sparsely distributed on the first segment. This type of sensilla occurs in the families Mesoveliidae (Fig. 4e) and Geriidae (Fig. 15f).
  3. *Sensilla basiconica 3 (SB3)* These are 5–18-µm-long pegs with a multi-porous surface, embedded in inflexible sockets. They are straight or bent towards the tip of the antenna. The stem of the sensillum is thick at the base and tapers upwards (Figs. 2, 18c). They are sparsely distributed on different segments either singularly or in groups—usually on the fourth antennal segment. This type of sensilla was observed in the families Mesoveliidae (Figs. 4f, g), Veliidae (Figs. 5d,e, 6e, 7c, d, 8c, 9f, h, 10i) and Gerridae (Figs. 11h, 14d).
  4. *Sensilla basiconica 4 (SB4)* These are 12–32-µm-long structures, straight, with a rounded tip that are embedded in inflexible sockets. The base of the sensillum is smooth and the rest of the stem is ribbed with a multi-porous system (Figs. 2, 18d). They occur on the fourth antennomer. This type of sensillum was observed in the families Hebridae (Fig. 13e) and Veliidae (Figs. 9g, 10f).
VIII. *Sensilla placoidea (SP)* These are flat or concave multi-porous plates (Figs. 2, 18e). They were found on the first and second segments. This type of sensilla was found in the families Hydrometridae (Fig. 16c) and Gerridae (Fig. 11h).
IX. *Sensilla bell-mouthed (SBm)* These are sensilla with a characteristic morphological structure—a funnel-shaped pit. It has a short sensory cone and is covered with a cuticular fold (Fig. 18f). They were found only in *Aquarius paludum* (family Gerridae) (Figs. 2, 14e).

## Discussion

Generally, the antennae of adult insects typically contain various types of sensilla that play important roles in a number of behaviours (Hu et al. 2009). This paper presents the first comparative morphology of the antennal sensilla in Gerromorpha and the SEM images provide information on the number, position and overall appearance of most of the types of sensilla. In the semiaquatic bugs, nine types of sensilla were observed on the antennae. Based on their morphological structures a putative function was established. Due to the fact, the function of the sensilla bell-mouthed (SBm) was not established, this type was not discussed. Moreover, we found a slight difference in sets of antennal sensilla among families of Gerromorpha.

Study of these sensilla in Gerromorpha was also very interesting due to their specific habitat, they live in more humid environments. So in the discussion below, a set of the antennal sensilla in semiaquatic and several terrestrial bugs have been compared, e.g. Pentatomomorpha, Scutelleridae (Romani and Rossi 2009), Pentatomidae (Silva et al. 2010; Zhang et al. 2014; Ahmad et al. 2016) and Coreidae (Gonzaga-Segura et al. 2013), Cimicomorpha: Miridae (Chinta 1997) and Reduviidae (Catalá 1997).

## Morphology and mechanoreception of sensilla

Insects are able to receive numerous different types of mechanical stimuli such as touch, air currents, sound, gravity and deformations of the body regions that are either caused by external forces or by self-generated movements (Keil 1997). Currently, in Gerromorpha, based on the morphological study, it can be stated that sensilla trichoidea (ST1, ST2, ST3), sensilla chaetica (SCh1, SCh2, SCh2), sensilla leaflike (SL), sensilla campaniformia (SCa) and sensilla basiconica (SB1) are responsible for mechanoreception. Because mechanosensilla can have numerous shapes and sizes (McIver 1975), several subtypes of mechanosensitive structures have been described in the present paper. We have observed that these sensilla usually densely cover the surface of the antennae except on the campaniforme sensilla.

Sensilla trichoidea and chaetica of different lengths and thickness are the most dominant mechanoreceptive sensilla types on the antennae in gerromorphan species, and in many other insects groups, which has been confirmed in different studies (Schneider 1964; Agren 1977; Chapman 1998). The base of these sensilla are embedded in a flexible socket for greater mobility (McIver 1975), so they can receive stimuli by being touched, moved or deformed. Some of the sensilla trichoidea are sensitive to the air movement (Shields 2010).

We estimated that, in gerromorphan species, two subtypes (ST1 and ST3) of sensilla are numerous and air sensitive, because they are long, probably flexible and can easily be subjected to the vibration of air waves. According to Gonzaga-Segura et al. (2013) some types of antennal sensilla in Coreidae (*Leptoglossus zonatus*) also function in this way. Presently we can state, that large striated sensilla trichoidea in *L. zonatus* are similar to the ST1 in gerromorphan species and small, smooth sensilla trichoidea in *L. zonatus* are also similar to ST3 in gerromorphan species. Two similar types of sensilla trichoidea were pointed in some species of Pentatomomorpha such as *Oncopeltus fasciatus* (Dallas) (Lygaeidae), *Lygaeus kalmii* Stål (Lygaeidae) and *Neomegalotomus parvus* (Westwood) (Alydidae) (Slifer and Sekhon 1963; Harbach and Larsen 1976; Ventura and Panizzi 2005). In Cimicomorpha, Chinta (1997) and Catalá (1997) straight sensilla trichoidea with different wall thicknesses were observed, which are similar to ST1 and ST3 of species in Gerromorpha.

However, among gerromorphan species the curved sensilla trichoidea (ST2) were specific to certain species (*Amemboa cristata, Amemboa brevifasciata, Onychotrechus esakii*) of one subfamily—Eotrechinae. We regard, based on their external structure, that these sensilla also receive air stimuli. Similarly, sensillum leaflike which occurs in some species of Gerromorpha (*Hydrometra stagnorum, Amemboa brevifasciata*) can receive signals in the same way as sensilla trichoidea.

Whereas, the function of sensilla chaetica in semiaquatic bugs may be involved in an initial recognition of the surface by touch (e.g. soft membrane or sclerite) of the host, as well as may support the antennae on different surfaces. These sensilla are less numerous than sensilla trichoidea, but they are more stout and present in most taxa except Gerrinae and Hydrometridae. However, in Hydrometridae, sensilla trichoidea can also respond to touch. Similarly, the shape and function of the antennal sensilla chaetica in some heteropterans was described by Catalá (1997), Chinta et al. (1997), da Rosa et al. (1999), Silva et al. (2010), Rani and Madhavendra (1995, 2005) and Gonzaga-Segura et al. (2013), who indicated that they have a mechanosensitive role, which enables true bugs to determine the position of their antenna with respect to their surroundings. Similarly, Keil (1997) mentioned that sensilla chaetica may allow detection and transmission of diverse mechanical stimuli. Bin (1981) suggested that some of sensilla chaetica can also perform a gustatory function, but they are usually stiffer than the other sensilla, tend to touch the substrate first and thus detect information directly from the host.

Another type of mechanical stimulation is represented by sensilla campaniformia and few sensilla basiconica. In Gerromorpha, sensilla campaniformia were found on the scape and pedicel, as well as on the flagellar segments of the antennae in the areas of the cuticle that are subject to stress. Their position, shape and structure evidently suggest a mechanosensitive function in the studied taxa. The same shape and size of sensilla campaniformia were described in some taxa of heteropterans such as Reduviidae: Triatominae (Catalá 1997), Coreidae (Gonzaga-Segura et al. 2013), in four species of pentatomids (Ahmad et al. 2016) and currently on the antenna of gerromorphan species. It seems that these sensilla are common in most taxa of Heteroptera. These sensory structures are considered to be strain detectors for other insects as well, which are stimulated by mechanical deformations of the cuticle by external forces or by self-generated movements (Pringle 1938; McIver 1975; Keil and Steinbrecht 1984; Gnatzy et al. 1987; Keil 1997; Chapman 1998). These sensilla have mainly been tested in many insects e.g. in cockroaches (Moran et al. 1971; Spinola and Chapman 1974; Zill and Moran 1981).

Similarly, sensilla basiconica with a smooth surface (SB1), are considered to be proprioceptive sensilla in four gerromorphan species (*Perittopus asiaticus, Gerris lacustris*, *Amemboa cristata*, *Rheumatogonus luzonicus*) In the study, these sensilla were visible and situated between two antennomers in order to receive signals regarding their location relative to each other. In other gerromorphan species, these sensilla were not visible but we assume that this is typical in remain species. This premise is based on other data because such function of the basiconic sensilla was also observed in many insects (Chapman 1998).

## Morphology and thermo-hygroreception of sensilla

According to the present study in Gerromorpha the thermo-hygrorecepion function is conducted by two subtypes of sensilla coeloconica (SCo1, SCo2), one type of sensillum ampulacea (SA) and sensillum basiconicum (SB2). Each of the sensilla were classified in the group of thermo-hygrosensilla, which consists of a non-perforated, thick-walled peg that is located in either a deep tube or a pit and is innervated by three neurons (Boo and McIver 1975; Altner et al. 1977; Altner and Loftus 1985; Shields and Hildebrand 1999). Usually, a sensilla coeloconica and ampulacea are less numerous than mechanosensilla and often appears singularly (Chapman 1998) which is confirmed in the present study.

Presently, in some species the coeloconic sensilla were identified as a larger opening (SCo2) in the subfamilies Mesoveliinae, Hebrinae, Ptilomerinae and Gerrinae in contrast to a smaller opening (SCo1) that is surrounded by finger-like structures (spines) in the subfamilies Veliinae, Perittopinae and Ptilomerinae. Moreover, the sensillum ampullacea described presently in some gerromorphan species (*Gerris lacustris, Aquarius paludum, Amemboa brevifasciata, Amemboa javanica, Onychotrechus esakii*), it is hidden further under the surface of the cuticle, and the pegs project into the long tube walls perpendicularly (pegs in tubes). Kleineidam (1999) suggested two hypotheses for the morphology of coeloconic sensilla and sensilla ampullacea, which may be in order to either save space on the surface of the antenna or to protect the sensory peg against environmental temperature fluctuations or against water loss. Both seem possible.

In this study was showed that the shape, place and number of sensilla coeloconica in Gerromorpha closely resemble those that have been observed in many other insects. This is evidenced by the presence the sensilla coleoconica [larger opening (SCo2)] on the antennae of some species in Pentatomidae, Pyrrohcoridae, Dinidoridae, Scutelleridae, Coreidae (Ahmad et al. 2016; Rani and Madhavendra 1995, 2005; Brézot et al. 1997; Silva et al. 2010; Gonzaga-Segura et al. 2013) as well as in fulgoromophans (Wang et al. 2012). It suggests the probable universality of these sensilla in Heteroptera and in other insects. The second subtype seems less common but it does not assume their absence in other taxa of Heteroptera.

Our data indicated sensilla ampullacea in five species of Gerridae (*Gerris lacustris, Aquarius paludum, Amemboa brevifasciata, Amemboa javanica, Onychotrechus esakii*); these sensilla were not found in the species of the other families of Gerromorpha. In contrast, sensilla ampulacea are frequently present on the antennae in other insects [e.g. in many hymenopterans, coleopterans, in mosquitoes (Boo and McIver 1975)]. Nevertheless, sensilla ampullacea in species from the family Kinnaridae (Hemiptea: Fulgoromorpha) were identified by Wang et al. (2012) and has been evidenced their presence in other infraorder of Hemiptera. In Gerromorpha the thermo-hygroreception is also conducted by non-porous sensilla basiconica (SB2), which were observed in *Mesovelia furcata* (Mesoveliidae) and in more advanced species, e.g. in *Metrocoris nigrofascioides* (Gerridae).

Although a combination of all four of the mentioned types of thermos-hygrosensilla were only present in the family Gerridae, each of them was individually found in different species. Only *Aquarius paludum* had s. ampullacea and s. coeloconica 2.

## Morphology and chemoreception of the sensilla

### Olfaction

The hairlike and other porous cuticular structures are essentially sensory devices through which chemical molecules may stimulate the receptor within the sensilla (Pophof 1997).

The quantity of molecules that is intercepted by a sensillum depends upon its surface area, as well as the number and location of the sensilla. The advantage of long sense organs (sensilla basiconica or trichoidea- hair) is a larger interception surface area. However, small sense organs (sensilla placoidea) are less exposed to chemical molecules than long sense organs (Lewis 1970).

In Gerromorpha, sensilla basiconica 3 (SB3), sensilla basiconica 4 (SB4) and sensilla placoidea (SP) are responsible for recognizing smell.

The porous, sensilla basiconica (SB3) in the studied species probably represent a single-walled pore sensilla type that was described by Slifer (1970). Furthermore, sensillum basiconicum 4, described in some gerromorphan species, belongs to multiporous, grooved, double-walled sensilla. Such types of sensilla were pointed by several authors (Slifer 1970; Zacharuk 1985; Cave and Gaylor 1987), and play an olfactory role by perceiving long-distance stimuli. The sensilla placoidea multiporous in some Gerromorpha are also similar to sensilla placoidea olfaction (Zacharuk 1980).

We observed that the different shapes and sizes of sensilla share common sensitivities in Gerromorpha. Based on present data, olfactory sensilla are not numerous in studied species. Olfactory sensilla are spread singularly and rarely on the surface of the antennae with the exception of *Pertitopus sp.* (Perittopinae, Veliidae) and *Microvelia douglasi* (Microveliinae, Veliidae), in which these sensilla are present numerously and in clusters on the last antennomer.

Interesting variations of olfactory sensilla were observed among representatives of families. The olfactory sensilla had a large degree of diversification of the sensillum types even within a species.

In representatives of Mesoveliidae (sensillum SB3), Hebridae (sensillum SB4) and Hydrometridae (sensillum SP), only one type of olfactory sensillum was identified, whereas in Veliidae (*Perittopus asiaticus* and *Velia caprai)* there were two types of sensilla (SB3, SB4). In turn, there were three types of sensilla (SB3, SB4, SP) in Gerridae (*Gerris lacustris*). We observed that in some species of Gerridae, the number of olfactory types of sensilla grew, but it is difficult to explain this phenomenon. We suppose that the sensilla basiconica subtype 4, plays an olfactory role on long-distance stimuli, while the other olfactory sensilla are probably responsible for short-distance stimuli.

In this respect, sensilla in Gerromorpha (*Gerris lacustris*) are not distinguished by the specificity of sensilla in other insects. For example, sensilla placoidea found in *L. zonatus* (Coreidae) (Gonzaga-Segura et al. 2013), are similar to sensilla placoidea in *Gerris lacustris*, sensilla basiconica subtype 3 are similar to sensilla basiconica SB4 in most gerromorphan and subtype 5 are similar to sensilla basiconica SB3 in gerromorphan. Three subtypes of basiconic olfactory sensilla were also observed in most of the other heteropteran species (Catalá 1997; da Rosa et al. 1999; Silva et al. 2010; Rani and Madhavendra 1995, 2005), which can be compared to the olfactory sensilla basiconica in gerromorphan. Also two different multiporous sensilla, i.e. short sensilla basiconica (SB) and sensilla placoidea (SP) in four pentatomid species (Ahmad et al. 2016) correspond to the same sensilla in Gerromorpha.

Moreover, Chinta et al. (1997) found multiporous trichoid sensilla with a thin cuticle on *L. lineolaris* and basiconic sensilla, which are similar to SB4 in gerrids, both suggesting an olfactory role.

So far, it has not been fully investigated why insect ORNs (olfactory receptor neurons) are housed in sensilla that have different shapes and sizes or how the sensillum structure affects the olfactory function (Shanbhag et al. 2000). Most insects have single-walled, double-walled blunt hairs—olfactory basiconic or placoid sensilla because functional specificity of sensilla seems to depend on the thickness of their wall (Zacharuk 1985; Shanbhag et al. 2000).

### Gustation

Gustatory sensilla are identified morphologically by the presence of a terminal pore and they are usually embedded in inflexible sockets (Altner and Prillinger 1980; Chapman 1998; Brożek and Zettel 2014). Internally they possess three to 10 neurons with unbranched dendrites, which extend along the length of the peg and terminate just beneath the pore (Altner and Prillinger 1980; Zacharuk 1985; Frazier 1985; Chapman 1998; Shanbhag et al. 2000). Based on the morphological characters of the antennal sensilla in Gerromorpha, sensillum trichoideum with a terminal pore (ST4) belongs to the group of chemosensilla (gustatory). In the present study, a single gustatory sensillum trichoideum (ST4) at the end of the last flagommer was observed in *Microvelia douglasi, Perittopus asiaticus*, *Velia caprai* and two of the same sensilla in *Strongylovelia philippinensis* (Veliidae). Our results cannot rule out the presence of such sensilla in all studied species of Gerromorpha because their location makes them difficult to observe and identify using SEM. However, it seems that the antennae do not play the most important role in recognizing the taste.

Similar results were observed in other heteropteran groups. Romani and Rossi (2009) in Scutelleridae (Pentatomomorpha) pointed a single sensillum basiconicum with a rounded tip and an apical pore which is similar to the sensilla classified as ST4 in this paper. Moreover, a singular sensillum trichiodeum on the antennae appears to be involved in chemoreception (gustation) as has been observed in many species of Pentatomomorpha (e.g. *Odontopus nigricornis* (Stål), *N. viridula* (L.) (Rani and Madhavendra 1995), *N. viridula* (L.) (Brézot et al. 1997) and Cimicomorpha *Lygus lineolaris* (Palisot de Beauvois) (Dickens et al. 1995). Moreover, Silva et al. (2010) described on the antennae two types of sensilla trichodea of both functions (mechano- and gustatory) in three species of Pentatomidae viz., *E. heros* (F.), *P. guildinii* (Westwood) and *E. meditabunda* (F.). According to other studies, taste is mainly recognized by the labial tip sensilla. It was documented by observing numerous gustatory sensilla in this area, in all families in Gerromorpha (Brożek and Zettel 2014) as well as in other hetropterans (Cobben 1978).

## Summary of the antennal sensilla among subfamilies of Gerromorpha

In this study, we observed different numbers of antennal sensilla as well as different morphological features of these sensilla in representatives of the families of Gerromorpha.

In Mesoveliidae, which, according to Andersen (1982) and Damgaard (2008, 2012) is the most basal family, we observed four subtypes of mechanosensilla (ST1, ST3, SCh2 and SCa), one thermo-hygrosensitive sensillum (SB2) and one olfactive sensillum (SB3). Sets of these sensilla provide their basal function of the antenna. These sensilla were not numerous and were infrequent on the antennae in the studied species of the family that belong to the base branch of the phylogeny of the Gerromorpha.

The least diverse sensilla (three subtypes—ST3, SCa and SB4) were observed in Hebridae and two different types of sensilla (SL and SP) were also found in Hydrometridae. However, we suggest that during the study, some types of sensilla were invisible in SEM, so it was difficult to determine the full set of antennal sensilla.

In the more evolutionarily advanced taxa such as Veliidae and Gerridae (Andersen 1982; Damgaard 2008), the specialisation of the types of sensilla are clearly visible and occurred numerously along the entire surface of the antennae. In fact, eleven subtypes of sensilla—ST1, ST3, ST4, SCh1, SCh2, SCh3, SCa, SCo1 and SB1, SB3, SB4—were observed in Veliidae. A dense distribution of ST and the presence of thick and long SCh were characteristic for this family. Moreover, only one subtype of sensillum coleolconicum SCo1 and two subtypes of olfactory sensilla basiconica (SB3, SB4) were visible.

Whereas in the Gerridae, which are considered to be the most specialized of the family, all of the sensilla subtypes were observed (ST1, ST3, SCh1, SCh2, SCa, SCo1, SCo2, SB1, SB2, SB3, SB4, SP, SA, SL and SBm) were observed in individual species. The dense distribution of ST was found, although it was not as dense as in Veliidae.

Sensilla leaflike SL was observed in the family Gerridae and also in the family Hydrometridae. These types of sensilla can be regarded as synapomorphy for these families. The presence of sensilla ampullacea in the Gerridae can be regarded as an autapomorphic character for this taxon.

When the antennal sensilla are compared to labial tip sensilla in Gerromorpha, there are some similarities in the types of sensilla. The labial tip sensilla that are similar to the antennal sensilla types, are the sensilla placoidea, sensilla basiconica and coeloconica. On the other hand, sensilla trichoidea, chaetica, leaflike, campaniformia, ampullacea and sensilla bell-mouthed were not found on the labial tip (Brożek and Zettel 2014).

## Conclusion

The antennal sensilla of Gerromorpha did not strongly differ from other species of Heteroptera, meaning that a similar pattern of antennal sensilla is visible. In both taxa the base group of sensilla consists of six main types of sensilla: trichoidea, chaetica, campaniformia, coeloconica, basiconica and placoidea. This could be evidence of an evolutionary trend in the development of the antennal sensilla in these groups. Only three types of antennal sensilla (leaflike, ampullacea and bell-mouthed) in Gerromorpha were different from other Heteroptera.

Judging by the morphology and function, the basal set of types/subtypes of the antennal sensilla is similar among families of Gerromorpha. However, in more evolutionary advanced families (Veliidae and Gerridae), additional types/subtypes of sensilla occur.

The presence of pores on sensilla basiconica and sensilla placoidea reflects the ability of antennae to perceive different chemical stimuli. The relatively higher abundance of different subtypes of mechanoreceptors should be considered as potential detectors for various mechanical functions. Furthermore, thermo-hygroreceptive sensilla of four various types in semiaquatic bugs did not strongly differ from terrestrial heteropteran species.

The gustatory sensilla were only present in one family (Veliidae); therefore, the antennae probably do not play the most important role in gustation.
